# Comparative Study on Growth Characteristics and Early Selection Efficiency of Hybrid Offspring of *Populus deltoides* ‘DD-109’ and *P. maximowiczii* in Liaoning, China

**DOI:** 10.3390/plants14010111

**Published:** 2025-01-02

**Authors:** Wei Liu, Chenggong Liu, Yan Zhang, Jinhua Li, Jiabao Ji, Xiaorui Qin, Fenfen Liu, Chengcheng Gao, Nairui Wang, Xueli Zhang, Ning Liu, Rusheng Peng, Qinjun Huang

**Affiliations:** 1 Liaoning Provincial Research Institute of Poplar, Gaizhou 115213, China; liuwei053@163.com (W.L.); zhangyan85913@163.com (Y.Z.); jjb0606@163.com (J.J.); xiaming@163.com (N.W.); 2 State Key Laboratory of Tree Genetics and Breeding, Research Institute of Forestry, Chinese Academy of Forestry, Beijing 100091, China; liucgwlq@163.com (C.L.); lijinh@caf.ac.cn (J.L.); lfflff0122@163.com (F.L.); gaocc@caf.ac.cn (C.G.); zxl961109@163.com (X.Z.); 3 Key Laboratory of Tree Breeding and Cultivation, State Forestry and Grassland Administration, Beijing 100091, China; 4 Shandong Territorial and Spatial Planning Institute, Jinan 250013, China; qinxiaorui@shandong.cn; 5 UGent-Woodlab (Laboratory of Wood Technology), Department of Environment, Ghent University, 9000 Ghent, Belgium

**Keywords:** poplar, direct and reciprocal cross progeny, quantitative mature age, path analysis, early selection efficiency

## Abstract

Poplar is an important tree species for timber supply and ecological protection in northern China. Cultivating and selecting high-quality varieties and germplasm resources suitable for cultivation are key factors in enhancing the quality and productivity of poplar plantations in the arid and semi-arid northern regions with shorter growing seasons. This study conducted a field cultivation experiment on 10 progeny clones from the direct cross (D × M) of imported *Populus deltoides* ‘DD-109’ with *Populus maximowiczii* and 7 progeny clones from the reciprocal cross (M × D) using one-year-old rooted cuttings planted at a 4 m × 8 m spacing. Based on 17 years of annual growth observations, the study systematically compared growth characteristics, age of quantitative maturity, path relationships between traits, and early selection efficiency in the hybrid offspring. The results indicated that the D × M population had superior diameter at breast height (DBH), tree height (H), and volume (V) compared to the M × D population, while the height-to-diameter ratio (HDR) was lower. The growth rate of the 17 clones peaked from 10 to 14 years, with annual volume growth increments (PAIs) higher than mean annual volume increments (MAIs) during the early growth stages; the quantitative maturity age ranged between 12 and 16 years. The D × M population generally reached quantitative maturity earlier than the M × D population, with the fastest clone maturing in 12 years. Four clones (DM-9-17, DM-9-18, DM-9-14, and MD-61) showed values for V, DBH, H, and HDR above the hybrid group average. Path analysis demonstrated that DBH had the most significant direct and indirect effects on V, suggesting it as the best predictor for V. Using DBH as a reference, correlation and early selection efficiency analysis showed a strong relationship between growth characteristics at planting years 4–5 and later-stage performance, indicating this as the optimal period for early selection. These findings contribute to evaluating the production potential of *P. deltoides* ‘DD-109’ and *P. maximowiczii* germplasm in northern China and provide valuable guidance for selecting poplar clones suitable for local cultivation, accelerating breeding processes, and informing management planning for poplar plantations.

## 1. Introduction

Poplar (*Populus* spp.), a genus in the Salicaceae family, is characterized by a moderate genome size, rich genetic diversity, and high phenotypic plasticity, making it a model tree species for studying the growth and development of trees and perennial woody plants [1,2]. Poplar is widely planted due to its fast growth, short rotation period, and broad adaptability, making it one of the primary fast-growing timber and pulpwood species globally [3]. With the continuous advancement of breeding technologies, many poplar hybrids have gradually been selected as excellent target species for biofuels and bioproducts, contributing significantly to the security of global timber resources and ecological sustainability [4,5,6]. Given these advantages and values, the collection, conservation, and evaluation of poplar germplasm resources play a crucial strategic role in ensuring forest biodiversity, maintaining ecosystem balance, promoting the development and utilization of forest resources, and alleviating the timber supply–demand conflict.

Northeast China is one of the three major forest regions in the country, covering a total forest area of 59 million hectares, accounting for 27% of the national forest area and 26% of the national timber volume. This region includes the timber forests and protective forests distributed across the three major mountain ranges of the Greater and Lesser Khingan, and Changbai Mountains, as well as the protective forests in the Northeast Plain, the eastern Inner Mongolia Plateau, and the Liaoxi Hills, collectively referred to as the “Northeast Forests” [7]. Poplar, as one of the key tree species in the “Northeast Forests”, holds an irreplaceable position in regional forest development and the sustainable management of forestry [5,8,9]. However, due to the shortening daylight hours in autumn and the prolonged low temperatures in spring in northern regions, coupled with relatively insufficient rainfall, the growing season of poplars is forced to shorten or halt prematurely, which weakens their fast growth, especially in young trees that are more sensitive to low temperatures [10]. Moreover, the current number of poplar cultivars in China is limited, with many low-yielding stands, low rates of improved varieties, serious seed fluffing, and insufficient new cultivars suitable for replacement [11]. Combined with global warming, frequent droughts, and restrictions on farmland use, the future development of poplar plantations in China will need to focus more on areas with poor conditions, arid and barren mountains, and cold regions [12]. However, at present, the number and types of adaptive varieties developed for these specific ecological and climatic zones are severely lacking. Therefore, from the perspectives of climate change and changes in land use for poplar cultivation, it is particularly urgent to explore and cultivate new fast-growing poplar cultivars with strong cold resistance and high regional adaptability to improve the overall quality of forests in northern China’s arid and cold regions and to ensure regional ecological security and timber production.

In tree improvement programs, considering both hybridization methods and clonal selection can maximize the enhancement of target traits and result in the development of ideal cultivars [13,14], with direct and reciprocal crosses being the most common and important mating methods in plant breeding [15]. For instance, the ‘NL895’ poplar, a hybrid of *P. deltoides* and *P. nigra*, exhibits characteristics such as rapid growth, robust resistance, high adaptability, and significant hybrid vigor in southern China [16]. Typically, some traits of hybrid offspring are significantly influenced by the maternal parent, or the maternal traits dominate in the genetic process, leading to a tendency for the hybrid offspring to resemble the maternal parent in certain traits, which results in different genetic expressions between direct and reciprocal crosses (maternal inheritance) [17]. This direct cross effectively uses the genetic characteristics of different parents and reveals the relationship between genotype and traits; reciprocal cross-breeding, by exchanging the roles of parents, can be used to analyze the role of parents in genetic transmission and determine whether traits are influenced by gender or whether there is a paternal inheritance effect, reflecting the contribution of the parents to the gene expression of the offspring [18]. Whether direct or reciprocal, the hybrid vigor exhibited by offspring not only helps to improve breeding efficiency and optimize breeding strategies but also plays a key role in revealing genetic mechanisms and understanding the inheritance patterns of traits [19]. In comparison to molecular breeding technologies, such as transgenics and gene editing, artificial cross-breeding remains the fundamental method for achieving hybrid vigor and developing new varieties of forest trees [14]. The primary reason for this is that molecular methods are predominantly conducted at the laboratory level, making it challenging to implement large-scale promotion and application in the field. In contrast, the materials generated through artificial cross-breeding can often be directly utilized in field production [20]. 

For poplar, most hybrid species grow faster and have stronger adaptability, making them highly promising for applications in the paper industry and bioenergy production [21]. By 2011, the area of hybrid poplar plantations in Europe had reached 9402 km^2^ [22], with the hybrid of *P. deltoides* and *P. nigra*, known as *P. euramericana*, being the most widely planted [23]. In addition to expanding planting areas, hybrid poplar breeding goals generally need to emphasize improvements in growth rate, adaptability, stress resistance, wood properties, tree form, and biomass allocation [24,25]. *P. maximowiczii* is a wild species of poplar from the section *Tacamahaca*, primarily distributed in the mountainous areas of Liaoning, Jilin, and Inner Mongolia in China, as well as Japan and North Korea [26,27]. It is highly regarded for its excellent cold tolerance and shade tolerance potential, and can be hybridized with several poplar sections to obtain desirable offspring [24,28,29,30]. However, although *P. maximowiczii* can be planted in frost-prone and high-altitude regions, and outperforms other clones in arid areas, its harvested wood yield and survival rate are significantly reduced [31]. In contrast, *P. deltoides*, native to the Mississippi River Valley in North America, is the most valuable species for cultivation within the section *Aegiros* [32], known for its exceptionally fast growth and strong ecological adaptability, as well as rich genetic diversity, making it a key parent species in poplar hybrid breeding [11,33]. However, most *P. deltoides* grow better in the water-abundant and relatively fertile soils of southern China than in the aridity, semi-aridity, and long winters of northern regions. Therefore, combining and recombining the excellent traits of these two species has biological and genetic potential, with the hope of cultivating the fastest-growing hybrid poplar species best suited to local environments.

In addition to selecting suitable hybrid parents, optimizing the quantitative maturity and breeding duration is also one of the key factors in improving breeding efficiency [34]. In forestry production and management, quantitative maturity age serves as a critical temporal indicator, frequently utilized as a basis for determining the optimal harvesting age. Harvesting trees at this stage can yield the maximum economic benefit in terms of timber production [35]. Predicting the quantitative maturity age of various forest tree species or clones at the earliest opportunity can inform the prioritization of tree species prior to afforestation. Traditional breeding cycles tend to be long, with the period from hybridization to the release of new cultivars (or reaching quantitative maturity) potentially taking several years or even decades [36], which severely limits practical production needs. Therefore, focusing on the target traits during the early stages of breeding allows for the early identification of promising individuals (or extreme phenotypes and genotypes) [37], thereby concentrating resources on these high-quality individuals, reducing ineffective screening processes, and shortening the cycle from breeding to commercialization [38,39]. Additionally, early selection improves breeding success rates and facilitates the rapid development of superior varieties with strong environmental adaptability [40]. For tree species with long breeding cycles, the final performance of early selection must also be checked for maturity performance in greenhouses or plantations, and further field production trials are required to verify the true phenotypic and genotypic performance [41].

Therefore, achieving suitable poplar cultivars for specific sites in the northeastern region of China is an urgent issue to address. The parental provenances in this study originate from different bioclimatic classifications [42]. *P. maximowiczii* demonstrates good cold resistance, while *P. deltoides* is characterized by rapid growth. Based on the above realities, we used *P. deltoides* ‘DD-109’ and *P. maximowiczii* as the main parents for conducting direct and reciprocal hybrid breeding experiments. The growth differences, quantitative maturity age, and early selection efficiencies of these two populations were systematically evaluated. We aimed to achieve three objectives: (i) to determine whether hybrid clones exhibit maternal genetic effects and growth difference; (ii) to evaluate whether direct (*P. deltoides* ‘DD-109’ × *P. maximowiczii*) and reciprocal (*P. maximowiczii* × *P. deltoides* ‘DD-109’) crosses have similar quantitative mature ages in the Liaoning region; and (iii) to identify the optimal year for the early selection of traits. In addition, this study not only reflects the growth adaptability of introduced poplar resources in specific regions of China from various perspectives, but also offers breeding resources that address the current challenges of insufficient quantity and low quality of poplar varieties in the country. Ultimately, the goal was to provide theoretical guidance for improving the local poplar production potential and germplasm resource reserves, accelerating the breeding process, and refining the harvesting management plan.

## 2. Results

### 2.1. Growth of Direct and Reciprocal Cross Populations

**Figure 1:**
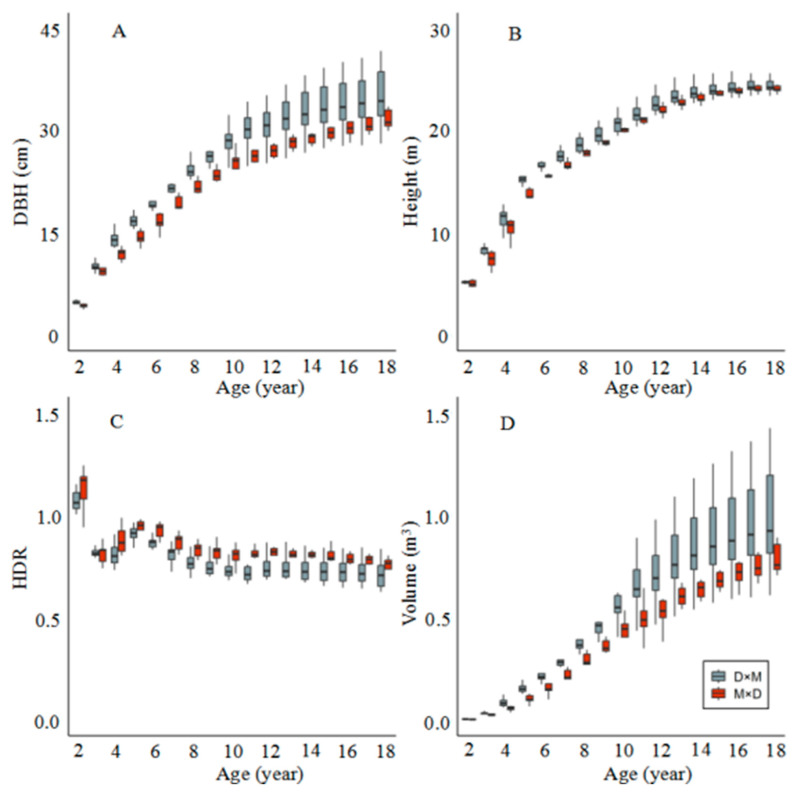
Growth traits of the D × M (*P. deltoides* ‘DD-109’ × *P. maximowiczii*) and M × D (*P. maximowiczii* × *P. deltoides* ‘DD-109’) populations. The letters (A, B, C, and D) in the figure represent the changes in DBH, H, HDR, and V of the hybrid populations with age, respectively.

**Figure 2:**
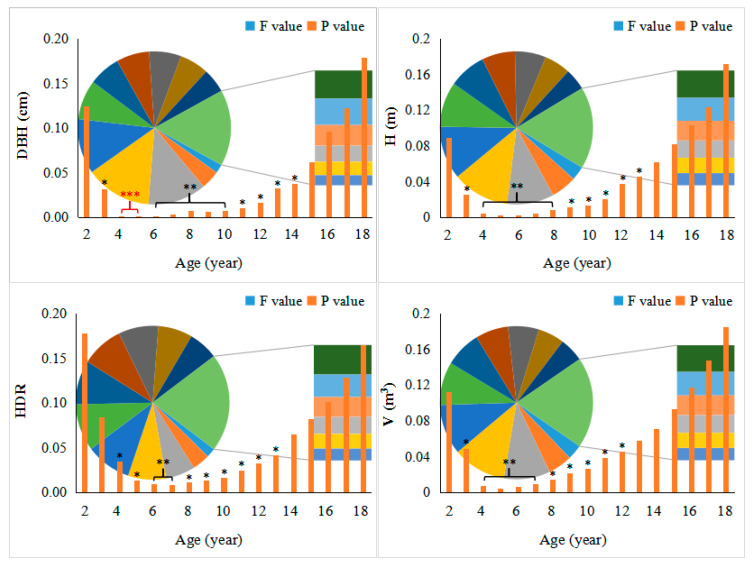
Analysis of variance between growth traits in the D × M and M × D populations. * indicates *p* < 0.05, ** indicates *p* < 0.01, and *** indicates *p* < 0.001.

Figure 1 shows the growth performance of the direct cross (D × M) and reciprocal cross (M × D) populations of *P. deltoides* and *P. maximowiczii* with increasing age. In general, all growth traits of the two populations were significantly different (*p* < 0.05) from year 4 to year 12 (Figure 2). After year 12, the significance gradually disappeared, which could be due to the greater variation in the D × M population and the lower mean of the poorer individuals. Moreover, the results showed that the diameter at breast height (DBH) values of the D × M population were always higher throughout the growth period, and significant (*p* < 0.05) differences appeared after the third year and disappeared after the fifteenth year (Figure 1A).

The results indicate that the D × M population consistently exhibited higher diameter at breast height (DBH) values throughout the growth period, particularly after the age of 10, with a more pronounced difference (Figure 1A). The height growth trends of both populations were similar, showing rapid growth initially followed by stabilization, but the height (H) of the D × M population was higher than that of the M × D population in most age groups (Figure 1B). The results for the height-to-diameter ratio (HDR) (Figure 1C) show that the HDR values for both populations gradually decreased with age and stabilized around 7 years old. Although there was little difference in the HDR between the two populations, the HDR of the M × D population was slightly higher in the early stages (2–6 years), indicating faster height growth at this stage. Additionally, the volume growth patterns both followed sigmoid tendencies (Figure 1D). These results suggest that D × M has an overall growth advantage, but also more accumulated variation in the later stages of the hybrid populations.

### 2.2. Differences in Quantitative Mature Age

**Figure 3:**
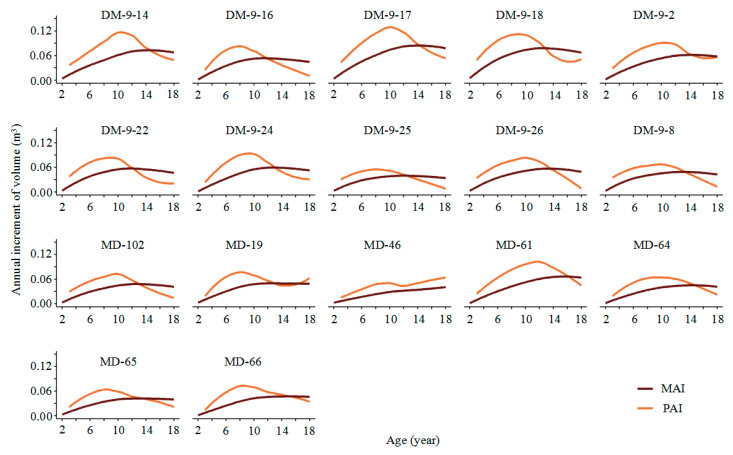
The MAI and PAI of clones. Clone prefix: DM represents direct cross offspring; MD represents reciprocal cross offspring.

Figure 3 shows that the mean annual increment (MAI) and the periodic annual increment (PAI) of most clones accelerate within a certain age range as they grow, reaching a peak before gradually slowing down. The peak age is generally between 8 and 12 years, after which both growth rates start to decline. The peaks and trends of MAI and PAI vary slightly among different clones. For example, clones like DM-9-16 and MD-65 reach their PAI peak at an earlier age, while clones like MD-61 and DM-9-2 experience a later peak. Some clones, such as DM-9-25 and MD-46, show MAI and PAI curves indicating lower growth rates than the other clones throughout the growth period. PAI is usually higher than MAI in the early stages of growth. As the trees grow older, PAI gradually decreases and eventually intersects with MAI, then mostly falls below MAI, indicating a reduction in the growth rate, with the cumulative growth amount becoming relatively stable.

Table 1 specifically displays the quantitative mature age (QMA) of different clones, and shows that there are significant differences in both maturity age and volume. The QMA of the D × M population is concentrated between 12 and 15 years, with clones DM-9-17 and DM-9-14 reaching maturity between 14 and 15 years and showing higher volumes (1.2 m^3^ and 1.1 m^3^, respectively). In contrast, the QMA of the M × D population ranges from 13 to 16 years, with most clones having smaller volumes at maturity, generally between 0.6 m^3^ and 0.7 m^3^. Notably, MD-46 had not reached quantitative maturity by the 18th year, indicating a slower growth rate for this clone. Overall, the direct cross combination shows greater potential for early maturity and volume accumulation.

### 2.3. Selection of Excellent Clones

**Figure 4:**
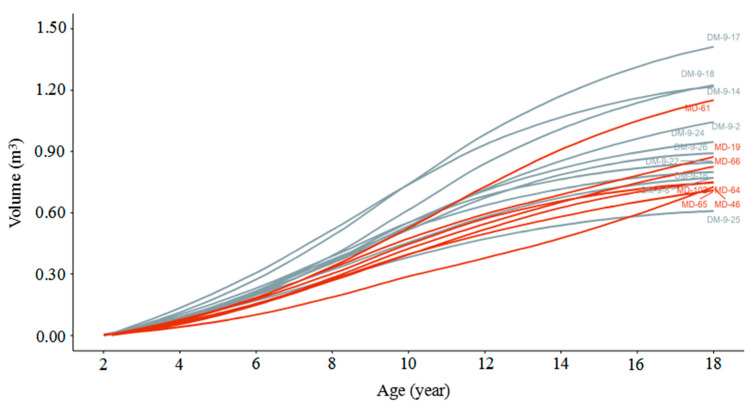
The variation trend of volume with age in different clones. Gray lines represent D × M acquired clones, and red lines represent M × D acquired clones.

As shown in Figure 4, at the same age, the volume of D × M individuals is generally superior to that of M × D individuals. The growth rate of the M × D curve is relatively lower, with most individuals having low volume levels. Within each population (D × M or M × D), there are also differences in growth among the different clones. In D × M, the clone DM-9-17 has the largest volume, followed by clones DM-9-18 and DM-9-14, with their volume growth rates also higher than other clones, while DM-9-25 is the smallest. In M × D, the clone MD-61 has the largest volume, with more noticeable growth in the later stages, while MD-46 and MD-65 show relatively slower growth.

As we are currently in the first generation of hybrid breeding, a slightly higher selection rate is warranted. Thus, we set it at 25%. Four clones were selected with a 25% selection rate, three of which came from D × M (DM-9-17, DM-9-18, and DM-9-14), and the other from M × D, MD-61. Table 2 shows that they all performed well within their respective hybrid populations, with the D × M having higher DBH, height, and volume compared to the M × D. Furthermore, DM-9-17 stood out in all traits, with particularly remarkable DBH (42 cm), height (26 m), and volume (1.4 m^3^), all far exceeding the average values of D × M, indicating that it is a very high-performing direct cross offspring. DM-9-18 and DM-9-14 also had a significant advantage in DBH and height compared to D × M’s average, and their volume (1.2 m^3^ each) was 20% higher than the group average, showing good growth traits. MD-61’s DBH (38 cm) and volume (1.2 m^3^) were significantly higher than M × D’s average, indicating that it is a reciprocal cross offspring with good growth potential. Generally, HDR reflects the morphological characteristics of trees; the lower the value, the more robust the trunk. The HDR values of the clones DM-9-17, DM-9-18, and DM-9-14 were lower than D × M’s average, and the HDR value of MD-61 was lower than M × D’s average, indicating that these clones have more robust and stable tree forms.

Volume variation reflects the degree of variability in volume within a population or among clones, indicating growth consistency and stability. The D × M population shows higher variability (26%) compared to the M × D population (19%), with individual clones exhibiting significantly lower variation, indicating improved growth uniformity. Clones such as DM-9-17 (13%) and MD-61 (5%) demonstrate the best performance in terms of both stability and potential for breeding applications (Table 3).

### 2.4. Path Analysis of Volume and Growth Traits

After conducting path analysis on the hybrid population’s volume, DBH, height, and HDR (Table 4), it was found that in all age stages and hybrid types, DBH had a significant positive direct or indirect effect on volume, indicating that DBH’s impact on volume is stable and significant, with the most pronounced direct and indirect effects observed at ages 7–9 years. In the D × M population, both the direct and indirect relationships between H and V were positive. In the M × D population, in the early growth stages (before 6 years), H had both positive direct and indirect effects on volume, with the indirect effect being more significant. However, from the 7th year of growth, the effects became negative. HDR had a positive direct effect on V, and this was significant or highly significant, except for the 2–3-year-old M × D population; the indirect relationship showed that, except for the 4–6-year age stage, HDR’s indirect effect on V was significantly negative. Furthermore, comparing different traits, it was found that both the direct and indirect effects of DBH on V were higher than those of H and HDR, with similar patterns observed in both reciprocal and direct crosses.

### 2.5. Early Selection Efficiency

The analysis of age–age correlations based on DBH was used to determine the effective early selection years (Table 5). The age of quantitative maturity was found to be between 12 and 16 years, with late maturity typically occurring around the 14th year. In terms of selection efficiency, although the D × M population showed the highest early selection efficiency (ESE) in the second year, the correlations between variables were not significant. In the third year, D × M showed good ESE and correlation (*R_p_*), but for the M × D population, the correlation was moderate (0.30 ≤ *R_p_* ≤ 0.50), suggesting that other variables might still have an impact. In the fourth and fifth years, the ESE remained high (≥1.93), and the correlations between variables were significantly strong (0.58 ≤ *R_p_* ≤ 0.71).

After the fifth year, although the *R_p_* values gradually increased and showed stronger significance, the ESE values decreased, indicating that while the accuracy of selection improved, the waiting period became too long. Therefore, using the growth performance in the fourth to fifth years as the optimal period for early selection can more accurately predict late-stage growth performance and aligns with the expected outcomes of early selection.

## 3. Discussion

For plants, hybrid offspring often exhibit superior phenotypes compared to any of the parent plants (i.e., hybrid vigor), with increased yield, improved seed quality, and enhanced resistance and tolerance to both biotic and abiotic factors [43,44]. Poplars are fast-growing and widely distributed tree species in the Northern Hemisphere, and have become the most intensively planted tree species in China’s commercial forests [12,33]. Under natural conditions, some species of the genus poplars are widely co-occurring, and extensive hybridization has been observed [45]. This hybridization is an important driver for genetic exchange, genetic variation, and evolution, as well as for the origin of new species and the genetic structure of populations [46]. Poplar breeders have discovered that excellent hybrid poplar varieties can be produced through artificial hybridization and strict breeding processes involving both foreign and local poplar species [24,47]. This hybrid vigor has created opportunities for developing tree species suited to short growing seasons and arid, cold, and nutrient-poor soil regions.

Among the various plant functional traits, tree height and diameter at breast height are critical indicators of plant growth, particularly in predicting stand productivity and tree volume [48,49]. Optimal tree height and diameter at breast height can serve as direct reflections of the quality of the current environmental site for the tree species [50]. Direct and reciprocal crosses are effective methods for assessing the potential of parental lines to produce superior clonal progeny [51,52,53]. Breeding practices have shown that, in the absence of cytoplasmic traits, phenotypic differences between offspring from direct and reciprocal crosses are minimal. However, when cytoplasmic inheritance is involved, significant phenotypic differences may arise between the two types of crosses [54]. In this study, through 17 years of growth observations of direct and reciprocal cross populations, it was found that the DBH and height of the D × M population were significantly higher than those of the M × D population. This result is similar to that of Pliura et al. [24]. It is speculated that this may be related to the broad ecological adaptability and strong growth vigor of *P. deltoides* [11,32], which promotes a more pronounced maternal genetic effect in the offspring [17,55], leading to the genetic characteristics of the maternal parent *P. deltoides* ‘DD-109’ combining with the cold resistance traits of *P. maximowiczii*, resulting in better longitudinal and radial growth of the trunk in the study area compared to the reciprocal cross population. Additionally, the superior clonal lines identified in the population (DM-9-17, DM-9-18, DM-9-14, and MD-61) exhibit significant genetic advantages, which may serve as new breeding parents. This would help breeders develop new cultivars with better adaptability and economic benefits, enriching the genetic resources of poplars and providing material for future breeding efforts.

Higher volume not only indicates greater timber yield but also suggests better economic benefits [56]. In this study, influenced by the combined advantages of height and DBH, the volume of the direct cross population was also greater than that of the reciprocal cross population, indicating that these poplars have higher production potential at maturity. Therefore, selecting hybrid combinations with larger volumes can significantly improve production efficiency and economic returns. Additionally, the tree’s HDR (height-to-diameter ratio) can be used to characterize its form and competitive effects [57], representing the mechanical stability and growth vigor of individual trees [58]. Numerous studies have shown that a lower HDR means that these trees are more robust and better able to withstand environmental stresses, such as strong winds and heavy snow [35,59,60]. In this study, the HDR of the D × M population was slightly lower than that of the M × D population, suggesting that trees in the D × M population have more stable structures and are better suited for regions requiring higher wind resistance and greater snowfall. On the other hand, the M × D population had a higher HDR, with thinner trunks, which might make them more vulnerable to damage from wind, rain, and ice during environmental stress [61,62]. This observation indicates that the D × M population exhibits greater resistance to wind and snow disasters, making it more suitable for cultivation in Liaoning, China. Therefore, when selecting sites for plantations, optimizing HDR through effective selection of forest breeding resources and forest management can ensure the long-term stability and timber quality of trees.

In addition to considering the growth phenotypes of trees, one of the most critical economic issues in forestry is the harvesting age of individual trees or stands [63]. Among these, the age at maximum sustainable yield (quantity maturity age) is an important method for determining the clear-cutting age. It is based on the theory of maximum sustainable yield, where the stand age at which the MAI equals the PAI is taken as the clear-cutting age [64]. In this study, the D × M population reaches quantity maturity earlier than the M × D population, indicating that the former (12–15 years) can achieve higher timber yields in a shorter rotation period, providing better economic returns, making it suitable for short-rotation industrial and pulpwood plantations. In contrast, the M × D population, with a later quantity maturity (13–16 years) and more stable volume growth, may be more suitable for long-term management of protective and ecological forests. The observed results are likely attributable to the distinct genetic characteristics of various hybrid groups or clones, as well as the differing interaction patterns between their genotypes and the local environment. Consequently, based on the specific forest land uses, producers can select appropriate hybrid combinations or clones that align with local climate characteristics to enhance afforestation objectives and improve forest stand management strategies [65].

However, as the growth age of trees increases, the difficulty and accuracy of growth measurements also increase. Therefore, selecting appropriate traits to predict difficult-to-obtain traits is extremely important. Studies have shown that path analysis can clearly indicate the direct or indirect effects between multiple traits, helping to assess the reliability of predicting one trait based on another [66]. In this study, we found that both in the direct and reciprocal crosses, DBH had a significant positive effect on volume, and its direct and indirect effects were greater than those of height and HDR, which is consistent with the findings of Wan et al. [67]. Compared to other growth traits like height, DBH is easier to measure and its measurement accuracy is easier to control [68]. Therefore, in future studies, DBH values of D × M population can be used to predict changes in volume, similarly to the conclusions of Rizvi et al. [69]. Additionally, in the process of tree trait evaluation, age–age correlation represents the consistency of tree traits across different growing years, which is an important indicator of the stability of a growth trait [70]. The juvenile–mature correlation of a trait or trait index is a key tool in forest genetics for calculating the gain from early-age selection [71]. If there is a strong correlation between juvenile expression and mature expression of a trait, it means that early testing and selection can achieve higher returns or improve genetic improvement rates [69]. The expected gain for each breeding cycle is smaller than the gain from directly selecting the target trait [72]. In this study, the DBH of hybrid populations showed a strong correlation of the 4th-5th years of growth, and later stages, with high early selection efficiency. This indicates that the excellent traits of the hybrids can be accurately predicted at this stage and continue into later growth stages. However, we also recognize that further consideration is needed to assess whether these traits are truly adapted to regions like Liaoning in China or areas with shorter, colder, or drier growing seasons, and the molecular mechanisms and biological characteristics behind these genetic adaptations still require further investigation.

## 4. Materials and Methods

### 4.1. Plant Materials

**Figure 5:**
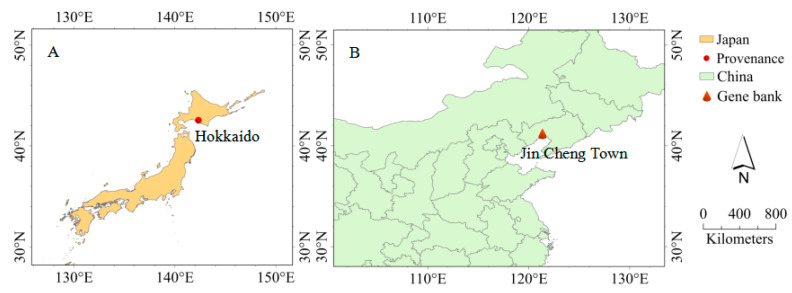
Distribution map of hybrid clones’ provenances and test sites. (**A**) shows the geographical locations of the hybrid clones in Hokkaido, Japan. (**B**) shows the geographical location of the test site in Jin Cheng Town, Linghai City, Liaoning Province, China.

**Figure 6:**
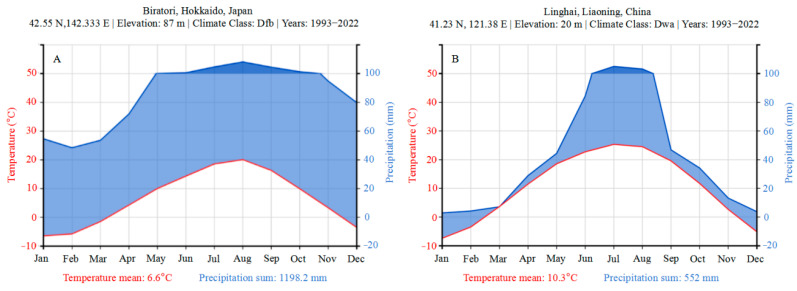
Temperature and precipitation of hybrid clones’ provenances (**A**) and test site (**B**). The red line represents mean temperature, and the blue line represents sum precipitation.

The experimental materials consisted of 23 hybrid clones, which were introduced from Hokkaido, Japan (42°33′ N, 142°20′ E, Figure 5A; the local temperature and rainfall changes are shown in Figure 6A [73]), to the Poplar Research Institute of Liaoning Province (41°61′ N, 122°41′ E) in 2002 through a resource exchange program. These hybrid clones were selected from two excellent F1 generation populations of *P. deltoides* ‘DD-109’ × *P. maximowiczii* and *P. maximowiczii* × *P. deltoides*. Three robust one-year-old stem segments, each stem segment 1.2 m in length and containing 14–19 dormant buds, were collected from each clone. Following a thorough evaluation by plant quarantine officials, which confirmed that the branches were free of diseases and insect pests, they were transported to their intended destination. In the spring of 2003, researchers conducted nursery propagation by cutting (cutting length of 15 cm). These cuttings were planted and cultured in plastic pots (30 cm in height, 28 cm in caliber) filled with substrate: 1 plant per pot, with 20 plants per clone. The substrate volume ratio was loess/charcoal/coarse sand = 5:1:1. The pH of the substrate was 6.73, the volume moisture content was about 46.78%, and the maximum moisture content was about 64.05%. Watering should be conducted once every 7 d during spring, every 3 d during summer, every 15 d during autumn, and once after winter. The volume of water applied each time was 1000 mL. From April to September, manual weeding and insect removal operations were performed every half a month.

### 4.2. Design and Site of Experiment

To maximize the number of hybrid clones planted in the field while retaining a sufficient number of seedlings for subsequent experiments and sustainable propagation, 17 clones (including 10 D × M clones and 7 M × D clones) with a survival rate greater than 60% were selected in mid-August 2004 (Table 6). In the fall of 2004, a plantation experiment was conducted using 1-year-old rooted cutting seedlings from these 17 clones. A gene bank was established to preserve these clones, with 8 healthy and pest-free plants per clone, characterized by straight stems. The planting density was 4 m × 8 m, and the experimental design followed a randomized block design with three blocks without soil heterogeneity. Each block contained two subplots (MD and DM), and the number of plants per clone in the three blocks was 3, 3, and 2, respectively. Weeds in the experimental forest are regularly cut and removed from June to July each year. Additionally, in the autumn of 2016, the dead branches on the tree trunks were cleared once.

The experimental plantation (gene bank) is located in Jin Cheng Town, Linghai City, Liaoning Province, in the Songnen Plain area of northern China (41°12′ N, 121°22′ E) (Figure 5B). This area lies in the eastern part of the Eurasian continent, in a warm temperate continental monsoon climate zone with distinct seasons. The groundwater table is 2–3 m deep, and the frost-free period ranges from 144 to 180 days. The temperature and rainfall variations over the past 20 years are shown in Figure 6 [73]. According to the World Soil Resources Reference Base (WRB) classification [74], the soil in the experimental forest is classified as thin humus calcareous meadow soil, with a texture ranging from sandy soil to sandy loam. The characteristics of the surface soil (0–20 cm) are as follows: pH 8.3–8.7, 1.19 g·kg^−1^ total N, 0.41 g·kg^−1^ total P, 10.58 g·kg^−1^ total K, 91.32 mg·kg^−1^ available N, 21.56 mg·kg^−1^ available P, 84.37 mg·kg^−1^ available K, 24.01 g·kg^−1^ organic matter, soil water content 17.33%, porosity 32.84%, and bulk density 1.70 g·cm^−3^.

### 4.3. Measurement and Analysis of Growth Traits

From the 2nd to the 18th year after planting, the tree height (H) and diameter at breast height (DBH) of all trees were measured annually in the fall using a Spiegel-Relaskop (Diangjiang Technology Co., Ltd., Shanghai, China) and a tape measure (YM-CL001, Yuma Tools Co., Ltd., Zhengzhou, China). Following the method of Konôpkr et al. [75], the height–diameter ratio (HDR) was calculated based on height and DBH, with the following formula:

$$\mathrm{HD}R=\frac{H}{\mathrm{DBH}}$$

The volume (V) was calculated according to the method of Peng et al. [76], with the following formula:

$$G_{1.3}=\pi \times {\left(\frac{\mathrm{DBH}}{2}\right)}^{2}$$

V = G_1.3_ × H × F_1.3_

where G_1.3_ is the chest height area; *π* is the parameter of the circumference ratio; DBH is the diameter at breast height; V is the trunk volume; H is the tree height; and F_1.3_ is the breast height form factor (F_1.3_ = 0.44, considered an estimate [77]).

### 4.4. Determining the Quantitative Maturity Age

By understanding the quantitative maturity age of various tree species, forestry operators can more accurately ascertain the optimal harvesting age. Based on the theory of maximum sustainable yield for forests, the traditional method for determining the quantitative mature age (QMA) is to take the forest age when the mean annual increment (MAI) of the forest volume equals the periodic annual increment (PAI) as the rotation age for final felling [35]. In simple terms, the year when the PAI curve intersects the MAI curve for each clone is the QMA. The formulas for calculating MAI and PAI are as follows:

$$\mathrm{MAI}=\frac{V_{t}}{t}$$

PAI = *V_t_*_+1_ − *V_t_*

where *t* is the age; *V_t_* is the volume of the year; *V_t+_*_1_ is the volume of the next year.

### 4.5. Path Analysis and Early Selection

Referring to the method of Wan et al. [66], path analysis was conducted using the path analysis module in SPSS software (Version 21, IBM Corp., Armonk, NY, USA) to analyze and compare the direct and indirect effects of DBH, H, and HDR on V under different hybrid methods and forest ages. Additionally, the correlation coefficient between each trait and the final year was calculated annually to determine the trait that best represents volume. Finally, early selection efficiency was calculated based on the method of Li and Wu [78].

Correlation coefficient (*R_p_*):

$$R_{p}=\frac{{CV}_{ij}}{\sqrt{{\delta_{i}}^{2}}\times \sqrt{{\delta_{j}}^{2}}}$$

In the formula, *R_p_* is the simple correlation coefficient between traits *i* and *j*, *CV_ij_* is the covariance of trait *i* with trait *j*, *δ_i_*^2^ is the variance of trait *i*, and *δ_j_*^2^ is the variance of trait *j*.

Path coefficient (DP):

$$\mathrm{DPC}=B_{i}\times \frac{S_{i}}{S_{y}}$$

IPC = *R_p_* × DPC

In the formula, *B_i_* is the partial regression coefficient of trait *y* on trait *i*, *S_i_* and *S_y_* are the standard deviations of traits *i* and *y*, DPC is the direct path coefficient of trait *i* on trait *y*, and IPC is the indirect path coefficient of trait *i* on *y* through trait *j*.

Early selection efficiency (ESE):

$$\mathrm{ESE}=R_{p}\times \frac{t_{e}}{t_{l}}$$

In the formula, *t_l_* and *t_e_* represent the early and late correlation ages, respectively.

## 5. Conclusions

This study systematically compared the growth traits of the hybrid populations of *P. deltoides* ‘DD-109’ and *P. maximowiczii* from both direct (D × M) and reciprocal (M × D) crosses. The D × M exhibited significant growth advantages in DBH, height, and volume, indicating the great potential for the application of direct hybrid poplar in fast-growing and high-yield plantations. The D × M had a lower HDR than the M × D, suggesting that the structure of the trees in the direct cross is more stable, while the reciprocal cross may face uncertainties in coping with environmental changes. The MAI and PAI of each clone increased at certain ages, with the growth rate accelerating within a specific age range, and PAI generally being higher than MAI in the early growth stages. The peak growth occurred between 8 and 12 years. The quantitative maturity age of the D × M poplars was found to be between 12 and 16 years, with the direct cross maturing earlier and accumulating more volume. In addition, with a 25% selection rate, the study identified four hybrid clones (DM-9-17, DM-9-18, DM-9-14, and MD-61) that exhibited optimal growth in volume and excellent DBH, height, and HDR. Based on the path analysis results, DBH had a greater direct and indirect effect on volume than height and HDR in both direct and reciprocal crosses, suggesting that DBH is the most reliable indicator for predicting the changes in volume. The study on the age–age correlation of DBH and early selection efficiency showed that the growth characteristics of the D × M poplars in the 4th to 5th year were significantly correlated with later performance, and had a high early selection efficiency, making it the optimal period for early selection. Although this study systematically analyzed the growth traits of hybrid poplars, superior growth phenotypes are the most direct indicators of a species’ environmental adaptability and stress resistance. However, for trees with longer growth cycles, the growth adaptability of hybrid poplars in the study area is a process that requires a comprehensive evaluation using multiple indicators. Therefore, future research should further clarify the biological mechanisms of drought resistance, disease resistance, and cold tolerance in the hybrid offspring to better carry out resource screening and conservation. Additionally, exploring the long-term performance of these hybrid combinations in diverse environments, and their comparative advantages and disadvantages with local native species, will help optimize breeding strategies and economic benefits.

## Figures and Tables

**Table 1:** Quantitative maturity of clones.

Clones	Quantitative Maturity	Volume (m^3^)	Clones	Quantitative Maturity	Volume (m^3^)
DM-9-22	12	0.7	DM-9-2	14	0.9
DM-9-16	12	0.6	DM-9-26	14	0.8
DM-9-18	13	1.0	MD-65	14	0.6
DM-9-24	13	0.8	DM-9-14	15	1.1
MD-19	13	0.7	MD-66	15	0.7
DM-9-8	13	0.6	MD-64	15	0.7
MD-102	13	0.6	MD-61	16	1.1
DM-9-25	13	0.5	MD-46	——	0.7
DM-9-17	14	1.2			

**Table 2:** Growth traits of the population and selected clones.

Hybrid Type/Clone	DBH (cm)	Height (m)	Volume (m^3^)	HDR
D × M populations	35	25	1.0	0.71
DM-9-17	42	26	1.4	0.62
DM-9-18	39	26	1.2	0.65
DM-9-14	39	25	1.2	0.63
M × D populations	32	24	0.8	0.75
MD-61	38	24	1.2	0.63

**Table 3:** Volume variation of the population and selected clones.

Hybrid Type	Volume Variation	Clone	Volume Variation
D × M populations	26%	DM-9-17	13%
		DM-9-18	6%
M × D populations	19%	DM-9-14	9%
		MD-61	5%

**Table 4:** The path relationships of volume with DBH, H, and HDR at different ages.

Hybrid Type	Age (Year)	DBH		Height		HDR	
		DPC	IPC	DPC	IPC	DPC	IPC
D × M	2–3	1.90 **	1.88 **	−0.57 **	−0.55 **	0.38 **	−0.34 **
	4–6	1.53 **	1.52 **	−0.61	−0.57 **	0.30 *	0.04
	7–9	2.07 **	2.05 **	−0.49 **	−0.45 **	0.74 **	−0.60 **
	10–12	1.51 **	1.48 **	−0.18	−0.15 **	0.49 **	−0.33 **
	13–15	1.66 **	1.65 **	−0.13 **	−0.11 **	0.60 **	−0.54 **
	16–18	1.63 **	1.62 **	−0.07 **	−0.06 **	0.59 **	−0.56 **
M × D	2–3	0.76 *	0.74 **	0.29	0.27 **	0.04	−0.03 **
	4–6	0.97	0.97 **	0.02 *	0.02 **	0.03 *	0.01
	7–9	2.28 **	2.26 **	−0.70 **	−0.66 **	0.75 **	−0.59 **
	10–12	1.57 **	1.55 **	−0.20 *	−0.15 **	0.55 **	−0.40 **
	13–15	1.59 **	1.59 **	−0.06 **	−0.04 **	0.56 **	−0.54 **
	16–18	1.51 **	1.50 **	−0.04 **	−0.03 **	0.49 **	−0.48 **

DPC represents the direct path coefficient, and IPC represents the indirect path coefficient. The asterisk (*) indicates a significant correlation (*p* < 0.05), and the double asterisk (**) indicates a highly significant correlation (*p* < 0.01).

**Table 5:** Analysis of early selection efficiency in hybrid populations.

Age (Year)	D × M		M × D	
	*R_p_*	ESE	*R_p_*	ESE
2	0.48	3.33	0.09	0.65
3	0.65 *	3.01	0.48 *	2.25
4	0.58 *	2.03	0.61 *	2.13
5	0.69 *	1.93	0.71 *	1.98
6	0.79 *	1.84	0.77 *	1.79
7	0.88 **	1.76	0.85 *	1.69
8	0.89 **	1.56	0.87 *	1.52
9	0.93 **	1.45	0.86 *	1.34
10	0.96 **	1.35	0.95 **	1.33
11	0.99 **	1.26	0.98 **	1.25
12	0.99 **	1.16	0.98 **	1.14
13	1.00 **	1.07	0.99 **	1.06

*R_p_* represents the age–age correlation coefficient, and ESE represents early selection efficiency. An asterisk (*) indicates a significant correlation (*p* < 0.05), and double asterisks (**) indicate a highly significant correlation (*p* < 0.01).

**Table 6:** Survival rate of 1-year-old clonal seedlings.

Hybrid Type	Clone	CN	SN	SR		Hybrid Type	Clone	CN	SN	SR	
D × M	DM-9-22	20	18	90%	≥65% (10 clones)	D × M	DM-9-15	20	8	40%	<60%
	DM-9-16	20	16	80%			DM-9-1	20	8	40%	
	DM-9-18	20	16	80%			DM-9-3	20	8	40%	
	DM-9-24	20	16	80%			MD-19	20	6	30%	
	DM-9-8	20	16	80%		M × D	MD-102	20	17	85%	≥60% (7 clones)
	DM-9-25	20	15	75%			MD-65	20	15	75%	
	DM-9-17	20	15	75%			MD-66	20	15	75%	
	DM-9-2	20	14	70%			MD-64	20	14	70%	
	DM-9-26	20	14	70%			MD-61	20	13	65%	
	DM-9-14	20	13	65%			MD-46	20	13	65%	
	DM-9-10	20	10	50%	<60%		MD-19	20	12	60%	
	DM-9-6	20	9	45%		Total	23 clones				

CN: number of cuttings; SN: number of surviving trees; SR: rate of survival.

## Data Availability

The data presented in this study are available upon request from the corresponding author. The data are not publicly available due to ethical reasons.
